# Les aspergillomes pulmonaires: à propos de 37 cas à Madagascar

**DOI:** 10.4314/pamj.v10i0.72209

**Published:** 2011-09-11

**Authors:** Joëlson Lovaniaina Rakotoson, Notahiana Razafindramaro, Jocelyn Robert Rakotomizao, Hanta Marie Danielle Vololontiana, Radonirina Lazasoa Andrianasolo, Kiady Ravahatra, Michel Tiaray, Jobeline Rajaoarifetra, Hendriniaina Rakotoharivelo, Ange Christophe Félix Andrianarisoa

**Affiliations:** 1 Unité de soins, de formations et de recherches de Maladies Infectieuses, du Centre Hospitalier Universitaire d'Antananarivo, Antananarivo, Madagascar

**Keywords:** Aspergillome pulmonaire, Aspergillus fumigatus, Prise en charge, Madagascar

## Abstract

L'aspergillome pulmonaire est une colonisation par *Aspergillus* d'une cavité pulmonaire préformée. Nos objectifs étaient de définir le profil épidémio-clinique et thérapeutique des aspergillomes pulmonaires et essayer de dégager les facteurs favorisants de cette affection à Madagascar. Nous avons réalisés une étude prospective, descriptive, analytique durant 59 mois sur les aspergillomes pulmonaires à Antananarivo Madagascar. Etaient inclus dans cette étude les malades ayant un diagnostic d'aspergillome pulmonaire. Trente-sept (37) cas d'aspergillome pulmonaire étaient recensés parmi les 8 392 patients hospitalisés dans le service de Pneumologie (0,44%). Il s'agit de 29 hommes (78,38%) et 8 femmes (21,61%), d’âge moyen de 43 ans. Les facteurs prédisposant étaient dominés par la tuberculose pulmonaire (89,19%). Le délai moyen d'apparition de l'aspergillome chez les malades ayant un antécédent de tuberculose pulmonaire à bacilloscopie positive (TPM+) était de 8 ans et 6 mois avec un délai extrême de un mois à 23 ans. L'hémoptysie était le mode de révélation le plus fréquent (91,89%). Le traitement était médical chez 27 patients (72,97%) et médico-chirurgical chez 10 patients (27,03%). Vingt sept patients étaient perdus de vue (72,97%), et pour les 10 patients suivis (27,02%), 70% avaient une évolution favorable avec disparition des signes, et 30% présentaient des hémoptysies récidivantes. Le taux de mortalité postopératoire était de 4% et 50% des patients avaient des complications postopératoires. La surveillance des lésions séquellaires de tuberculose pulmonaire qui constituent les facteurs favorisants prédominant d'aspergillome pulmonaire à Madagascar nécessite une attention particulière. La prise en charge de la tuberculose doit être précoce et adaptée surtout dans les pays à forte prévalence tuberculeuse.

## Introduction

Les aspergilloses sont les infections mycosiques pulmonaires les plus fréquentes. L'aspergillome correspond à une prolifération de spores aspergillaires organisés en un feutrage dense au sein d'une cavité préformée pulmonaire. Les auteurs rapportaient une étude sur les aspergillomes pulmonaires à Madagascar afin d’établir les particularités épidémio-cliniques et thérapeutiques de cette affection, et d'en déterminer les facteurs favorisants.

## Méthodes

Nous avons réalisés une étude prospective, descriptive et analytique des aspergillomes pulmonaires durant une période de 59 mois (du janvier 2006 au novembre 2010) dans le service de Pneumologie du CHU d'Antananarivo Madagascar. Ont été inclus dans cette étude les patients présentant une forte suspicion clinico-radiologique d'aspergillome associée ou non à une sérologie aspergillaire et un prélèvement mycologique positifs. Les données épidémiologiques (âge, sexe, antécédents, facteurs favorisants), cliniques (mode de révélation, signes cliniques), paracliniques (sérologie aspergillaire, radiographie et scanner thoracique), thérapeutiques et évolutifs étaient étudiées.

## Résultats

Trente sept cas (37) d'aspergillome pulmonaire étaient recensés parmi les 8 392 patients hospitalisés dans le service de Pneumologie (0,44%). Il s'agit de 29 hommes (78,38%) et 8 femmes (21,61%), d’âge moyen de 43 ans. Concernant les facteurs de risque, parmi les 37 patients 4 seulement n'avaient pas d'antécédent de tuberculose (11%). Les 33 autres patients étaient des anciens tuberculeux (89,19%) parmi les quels 31 (83,78%) avaient une tuberculose pulmonaire à microscopie positive (TPM+) et 2 patients (5,4%) une tuberculose pulmonaire à microscopie négative (TPM-). Six cas de bronchopneumopathie chronique obstructive (16,21%), 2 cas d'abcès pulmonaire (5,4%) et un cas de dilatation des bronches (2,7%) avaient été individualisées. Les autres facteurs de risques sont donnés par le Tableau 1. Le délai moyen d'apparition de l'aspergillome chez les malades ayant un antécédent de TPM+ était de 8 ans et 6 mois avec un délai extrême d'un mois à 23 ans. Chez les 2 patients ayant un antécédent de TPM-, ce délai était respectivement de 7 ans et 30 ans. Les motifs fréquents d'hospitalisation étaient dominés par l'hémoptysie (91,89%), la toux (46%) et la fièvre (35%). La radiographie du thorax réalisée chez 32 patients montrait des images de caverne dans 13 cas (40,6%), de grelot dans 14 cas (43,7%), de masse pulmonaire dans 3 cas (9,3%), de nodule dans 2 cas (6,2%) (Tableau 2). Le scanner thoracique réalisé chez 19 patients montrait des images de cavernes dans 10 cas (52,6%), de grelot dans 7 cas (36,8%), de masse et de nodule dans chacun 5,2% de cas (Tableau 2). Sur le plan biologique, tous les 6 patients (16,21%) bénéficiant d'une sérologie aspergillaire avaient un résultat positif. Par contre aucun examen mycologique et histologique n'a été effectué. Le traitement était médical chez 27 patients (72,97%) utilisant l'Itraconazole 200 mg par jour pendant 6 mois, et médico-chirurgical chez 10 patients (27,03%) (Lobectomie ou pneumonectomie). Une perte de vue de 17 patients parmi les 27 traités médicalement (62,96%) a été constatée. Parmi les 10 patients suivis (37,04%) 7 avaient une évolution favorable (25,92%) avec disparition des signes cliniques et amélioration des images radiologiques; 3 avaient des hémoptysies récidivantes et une stabilité des lésions radiologiques (11,11%). Parmi les malades sous traitement médico-chirurgical, 50% avaient une surinfection pleuro-pulmonaire conduisant à une pleurostomie. Le taux de mortalité postopératoire était de 4%.

**Tableau 1:** Les facteurs de risque présents chez 37 patients pris en charge pour aspergillose pulmonaire au Centre Hospitalier Universitaire d'Antananarivo, Madagascar

Facteurs de risque		Effectif	Pourcentage
**Tuberculose**		33	89,1
	TPM+	31	83,7
	TPM-	2	5,4
**Autres atteintes pulmonaires**		**9**	**24,3**
	Abcès	2	5,4
	BPCO	6	16,2
	DDB	1	2,7
**Alcoolo-tabagisme**		**18**	**48,6**
	Alcool	7	18,9
	Tabac	3	8,1
	Alcool et tabac	8	21,6
**Immunodépression**		**2**	**5,4**
	VIH	1	2,7
	Cancer	1	2,7
**Exposition aux polluants**		**12**	**32,4**
	professionnels	7	18,9
	domestiques	5	13,5

TPM+: tuberculose pulmonaire à bacilloscopie positive, TPM-: tuberculose pulmonaire à bacilloscopie négative, BPCO: bronchopneumopathie chronique obstructive, DDB: dilatation des bronches

**Tableau 2:** Trouvailles radiologiques chez 37 patients pris en charge pour aspergillose pulmonaire au Centre Hospitalier Universitaire d'Antananarivo, Madagascar

Imagerie	Radiographie du thorax		Scanner thoracique	
	Effectif n= 32	pourcentage	Effectif n= 19	pourcentage
Cavernes	13	40,6	10	52,6
Image en grelot	14	43,7	7	36,8
Masse pulmonaire	3	9,3	1	5,2
Nodule	2	6,2	1	5,2

## Discussion

Les lésions séquellaires de tuberculose constituent les facteurs prédisposant les plus fréquents d'aspergillome pulmonaire dans les pays à forte endémicité tuberculeuse comme Madagascar. Ces lésions peuvent être des cavernes et ou des bronchectasies. Quatre vingt neuf pourcent de nos malades avaient un antécédent de tuberculose pulmonaire. Mais d'autres facteurs constitués par les lésions de sarcoïdose [1–4], de kyste hydatique [5], de cancer bronchique excavé, de l'infarctus pulmonaire, de l'abcès pulmonaire, de la fibrose apicale d'une spondylodiscite ankylosante, du pneumothorax spontané et de l'emphysème bulleux [2] ont été rapportés. Le délai moyen d'apparition de l'aspergillome après la tuberculose pulmonaire est variable allant de quelques jours à plusieurs années. Il était dans notre étude de 8 ans et 6 mois avec un délai extrême d'un mois à 23 ans. La présentation clinique la plus fréquente est l'hémoptysie bien que la forme asymptomatique soit fréquente [6]. Le saignement provient de la sécrétion d'endotoxines par *Aspergillus* et ou de l'irritation mécanique des vaisseaux par la truffe aspergillaire mobile à l'intérieur de la cavité [7]. Le taux de mortalité dû à l'hémoptysie varie entre 2 à 14% [8]. Sur le plan radiologique, l'aspergillome se présente typiquement comme une masse intracavitaire, mobile, surmontée d'un croissant gazeux apical appelée « grelot aspergillaire ». Un épaississement de la plèvre adjacente est habituellement associé. La modification d'une cavité préexistante peut être le premier signe radiologique [9].

Il n'y a pas de consensus sur le traitement de l'aspergillome [6]. Cependant, les recommandations d'expert américaines suggèrent l'utilisation de l'Itraconazole pour le traitement des aspergillomes complexes (plusieurs cavités à parois épaisses, mal circonscrites au sein d'un parenchyme pulmonaire remanié: forme frontière avec l'aspergillose chronique nécrosante), soit en périopératoire, soit de manière exclusive chez les patients inopérables [10]. L'abstension thérapeutique est la règle notamment chez les patients asymptomatiques. Le traitement est entrepris seulement chez les malades symptomatiques et ou présentant une hémoptysie [6]. L'utilisation d'antifongiques par voie locale, endobronchique ou intracavitaire, a montré son efficacité sur de petites séries de patients inopérables [11]. L'instillation percutanée d'Amphotericine B guidée par le scanner thoracique peut être utilisée en cas d'hémoptysie avec arrêt de celle-ci en quelques jours [6]. L'Itraconazole orale est la molécule de choix en cas d'aspergillome pulmonaire du fait de sa bonne pénétration tissulaire [6]. Mais il n'existe pas à l'heure actuelle de preuve de l'efficacité des traitements antifongiques [12]. La résection chirurgicale de la cavité et de la truffe aspergillaire est habituellement indiquée en cas d'hémoptysie et chez les patients ayant une fonction respiratoire satisfaisante [6]. Ce type de traitement est difficile et dangereuse, émaillé de complications. Il est associé à un taux de mortalité élevé allant de 7 à 23% [13] selon les études. La morbidité peut atteindre 32%: hémorragies, défauts de ré-expansion pulmonaire, fistules broncho-pleurales, empyème [14]. La spéléotomie reste un traitement de dernier recours. L'embolisation par angiographie sélective est une alternative de temporisation en cas d'hémoptysie menaçante [14]. Dans son travail sur 278 traitements chirurgicaux d'aspergillome pulmonaire, Caidi et al [15] ont constaté 16 décès postopératoires (5,7%), des complications à type d'empyème (12,5%), une réexpension incomplète pulmonaire (9,3%), un saignement postopératoire (5%), des infections respiratoires (4,6%), une insuffisance respiratoire (4%), une fistule bronchique (2,5%). Guerra et al [16] retrouvaient un taux de mortalité postopératoire de 5% et de complications postopératoires de 16,3% dans son étude sur 60 cas d'aspergillome. Dans notre série, nous avons réalisés un traitement médical seul dans 72,9% de cas et un traitement médico-chirurgical dans 27,03% des cas avec un taux de mortalité postopératoire de 4% et 50% de surinfections pleuro-pulmonaires postopératoires. Mais notre étude était marquée par un taux de perdu de vue élevé (62,9%) surtout pour les malades traités médicalement, rendant ainsi difficile l'appréciation de l’évolution des ces patients. Ceci pourrait être expliqué par des problèmes pécuniaires devant le coût prohibitif des examens paracliniques et des médicaments à la charge des malades en totalité. Selon son étude sur 72 patients, Sagan et al [17] ne retrouvaient pas de bénéfice sur le traitement adjuvant par un antifongique à un traitement chirurgical. Ils ont même conclu la présence de nombre élevé d'effets secondaires de cette molécule.

## Conclusion

Nous insistons sur le dépistage et le traitement précoce et adéquat de la tuberculose pulmonaire dans les pays à forte prévalence tuberculeuse ainsi que la surveillance régulière des lésions pulmonaires séquellaires. Le traitement de référence de l'aspergillome qui est la chirurgie est grevé d'une lourde mortalité et de complication postopératoire.
